# Evaluation of the Effect of Fly Ash on Hydration Characterization in Self-Compacting Concrete (SCC) at Very Early Ages Using Piezoceramic Transducers

**DOI:** 10.3390/s18082489

**Published:** 2018-08-01

**Authors:** Yu Zheng, Dongdong Chen, Lingzhu Zhou, Linsheng Huo, Hongwei Ma, Gangbing Song

**Affiliations:** 1 School of Environment and Civil Engineering, Dongguan University of Technology, Dongguan 523808, China; zhengy@dgut.edu.cn (Y.Z.); mahongwei@dgut.edu.cn (H.M.); 2 Key Laboratory of Coastal and Offshore Engineering, Dalian University of Technology, Dalian 116024, China; chendongdlut@mail.dlut.edu.cn (D.C.); lingzhu_zhou@163.com (L.Z.); 3 Smart Materials and Structures Laboratory, Department of Mechanical Engineering, University of Houston, Houston, TX 77204, USA

**Keywords:** self-compacting concrete (SCC), fly ash, piezoceramic transducers, smart aggregates, hydration, wavelet packet energy analysis

## Abstract

Nowadays, the industrial waste, Fly Ash (FA), as a mineral admixture or a replacement of cement for the production of self-compacting concrete (SCC) has been increasingly used, because of its benefits in enhancing both fresh and long-term concrete properties and in promoting environmental-friendly construction. In this study, the conventional cement was replaced by FA at different rates (0%, 20%, 40%, 60% of the cement mass) for the SCC mixtures. The early-age (0–24 h) SCC hydration, which is a complicated chemical reaction in pozzolanic behavior, was characterized by using a pair of piezoceramic Smart Aggregates (SAs). One SA works as an actuator and the other works as a sensor. A sweep sine signal from 100 Hz to100 kHz was used as the excitation signal, which is helpful to understand the quantitative influence of fly ash on the kinetics of SCC hydration. During the hydration reaction, the received electrical signal was continuously detected by the sensor. The experimental results showed that increasing the volume of fly ash resulted in longer pozzolanic reaction time in SCCs, which successfully reveals the effect of fly ash volume on the hydration behavior in early age (0–24 h) hydration. In order to quantitatively evaluate the hydration in the 0–24 h, based on the wavelet packet energy analysis, the hydration completion index (HCI) and normalized hydration completion index (NHCI) were defined. The experimental results showed that the NHCI can clearly reveal the hydration completion progress during the early hydration age (0–24 h). To validate the accuracy of the test results based on SAs, a series of mechanical tests for penetration resistance of SCCs with different volumes of fly ash were carried out. The results predicted by the signal based on SAs gave reasonable agreement with the test results of penetration resistance. It can be concluded that a successful investigation of the influence of fly ash on early-age SCC hydration response can be achieved based on the analysis of the received electrical signal using the proposed method and the important hydration characteristics, such as initial and final setting time, and can be approximately predicted by NHCI values.

## 1. Introduction

**Figure 1:**
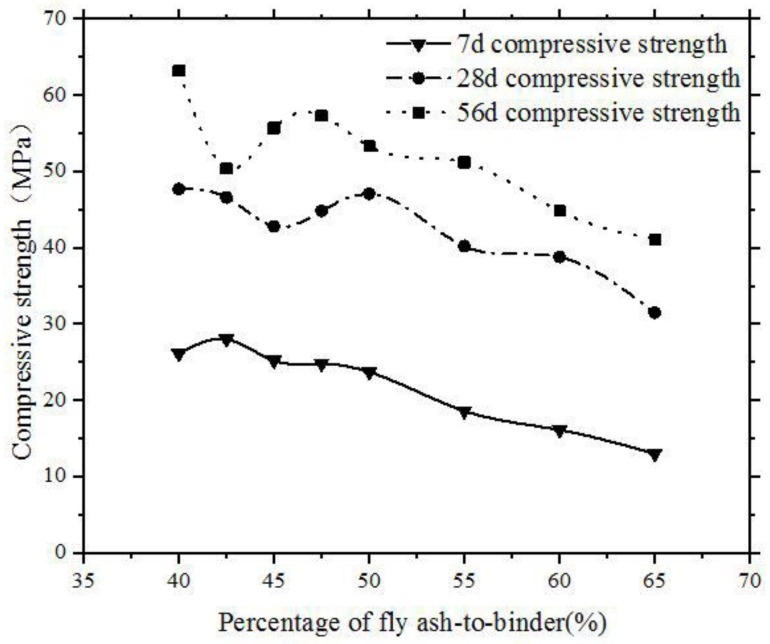
Influence of volume of fly ash on the compressive strength of SCC at different ages [8].

Self-compacting concrete (SCC) is a new category of high performance concrete characterized by its ability to spread into place under its own weight without vibration, and to self-compact without bleeding and segregation [1]. Currently, high-volume fly ash, as much as 50%, is used to replace the cement in the self-compacting concrete. The high-volume fly ash helps to achieve good slump flow of the SCC. Using fly ash in SCC is beneficial to improve workability and flowability, as the incorporation of continuously graded cementitious materials and fillers reduces interparticle friction [2,3]. Also, the application of fly ash improves rheological properties and reduces cracks in concrete due to the lower heat of hydration with lower amounts of Portland cement in the SCC mixing [4]. The significant use of fly ash as a cement replacement for SCC production opens the possibility for more sustainable concrete. The interests of developing more environment-friendly concretes where cement consumption is reduced using fly ash as cement replacement materials justifies the further exploration of the role this mineral material plays in concrete performance. The mechanical properties, durability and fresh states of SCC with high-volume fly ash have been investigated by many researchers [5,6,7]. It has been revealed experimentally by the co-authors that the strengths of SCC with high-volume fly ash were lower than that of concrete with pure Portland cement, especially at early age, as shown in Figure 1 [8]. This could be because of the dilution effect and low pozzolanic reaction [6,9,10]. It has been well reported in the literatures that the strength of hardened concrete is mainly determined by the curing during the SCC hydration process [7]. By using isothermal calorimetry, thermogravimetry (TGA), X-ray diffraction (XRD), scanning electron microscopy (SEM) techniques, and pore solution analysis, Weerdt et al. [11] studied the effect of minor additions of limestone powder on the properties of fly ash blended cements. Weerdt et al. also investigated the interaction between limestone powder and fly ash in ternary composite cement [12]. Berry et al. described the investigations of high-volume fly ash (HVFA)-Portland cement (PC) binders, the physical and chemical properties of which have been characterized up to 365 days of curing [13]. Although there are plenty of studies about the mechanisms of hydration reactions. The monitoring of the effect of fly ash on hydration characterization in concrete at very early ages is still rather limited. Therefore, it is necessary to investigate the effect approach to monitor the characterization of mineral material on the hydration process.

Until now, many researchers have carried out research on early-age concrete hydration monitoring to reveal this fundamental process of concrete settling and hardening. The temperature measurement is a traditional method to monitor early-age concrete hydration [14]. Optic fiber sensors, infrared thermography technology, and thermal couples were used to measure the temperature variations during the hydration process and the concrete early-age strength development can be estimated based on the measured temperature information [15,16]. Apart from the temperature measurement, wave propagation-based concrete hydration monitoring has also received attention [17,18]. Ultrasonic wave measurement is a popular method to reflect the change of concrete properties due to the high sensitivity of the ultrasonic testing method to microstructural changes in materials [19]. Lead Zirconate Titanate (PZT) is widely used [20,21] to generate and detect the ultrasonic waves because of its strong piezoelectric effect [22,23]. Based on PZT, two major approaches, the electromechanical (E/M) impedance [24] and the active sensing method [25], were often used.

Impedance of PZT is very sensitive for the changes of concrete properties [26,27]. A re-usable PZT transducer setup for monitoring the initial hydration of concrete and structural health was developed in Reference [28]. The influence of setting and initial hardening of concrete were investigated to detect the gradual bonding between a steel reinforcing bar and fresh concrete [29]. With the help of fuzzy logic [30], the strength development process of a high-strength concrete was monitored. An artificial neural network algorithm to estimate the early-age strength of concrete based on the EMI dynamic response of PZT sensor was proposed [31]. A wireless monitoring system which combined with the EMI approach was proposed by Kim et al. [32].

On the other hand, an active-sensing approach using a pair of piezoceramic smart aggregates (SAs) was first proposed by Song et al. [33] for concrete structural health monitoring. Smart aggregates (SA) were also utilized to perform structural health monitoring of a reinforced concrete (RC) bridge column subjected to pseudo-dynamic loading by Kong et al. [34]. Kong et al. also proposed a comparative study of the very early age cement hydration monitoring using compressive and shear mode smart aggregates using the active sensing method [35]. A support vector machine (SVM) was used to classify the concrete strength in the research by Kim el al [36]. Based on the velocity change, early-age concrete strength can also be evaluated [37,38,39]. A swept sine wave and several constant frequency sine waves were produced in Reference [40] to completely understand the hydration condition of the inhomogeneous, over-cluttering, and high-scattering characteristics of concrete.

However, the influence of high-volume fly ash on the very early-age hydration performance of SCC has not received enough attention in the existing literature. It is well known that concrete hydration at a very early age (0–24 h) plays a significantly important role in the entire hydration process and the concrete experiences a complicated chemical reaction from the liquid stage to the hardened stage after concrete casting. In the study by Zhu et al., piezoceramic bender elements were utilized to measure the velocities of the P-wave and S-wave to determine the hydration of concrete paste successfully during the first 6 h after casting [41]. Until now, little research has been conducted on the use of continuous testing with piezoceramic transducers on hydration performance of SCC with high-volume fly ash.

Therefore, the authors applied an active-sensing approach using SAs based on piezoceramic transducers to monitor very early age hydration characteristics of SCC with different volumes of fly ash. A pair of SAs were embedded in the test specimens before concrete casting. In the hydration process, one SA which works as an actuator to emit the sweep sine signal (100–100k Hz), and the other one works as a sensor to detect the propagated wave.

The evolution of hydration development gives a complete view on the complicated chemical reaction between the binder (cement and active mineral material) and water, and allows the determination of the effects of fly ash on hydration kinetics. Three states during SCC hydration were found: (1) liquid; (2) transition; and (3) solid. The monitoring results were compared with the mechanical test of penetration resistance of SCC and mortar. By comparing the results from SAs and penetration resistance, the meaningfulness and feasibility of this test method based on SAs were assessed in the case of hydration characteristics of SCC with high-volume fly ash.

## 2. Smart Aggregate Based Active Sensing Approach

**Figure 2:**
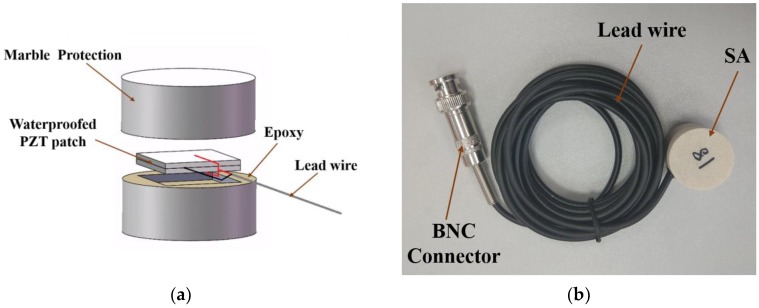
Smart Aggregate (SA): (**a**) The design illustration of a smart aggregate; (**b**) the photo of a smart aggregate.

The design schematic of smart aggregate (SA) is shown in Figure 2a. The core of the SA is a pair of bonded PZT patches that share common electrodes. The poling directions of the two PZT patches are against each other. In between the two PZT patches is a shared thin copper film electrode that is bonded to both patches using conductive epoxy. Wrapped outside of the two PZT patches is a thin copper film that serves as an electrode and as an electromagnetic shield. This thin copy film is bonded to the PZT patches using conductive epoxy. Lastly, these two PZT patches with electrodes are applied with a layer of nonconductive epoxy and are sandwiched by two mating marble blocks to provide the protection. The outer copper thin film provides an electromagnetic shielding effect to the PZT patches, and inner nonconductive epoxy provides waterproof. Finally, the marble blocks provide mechanical protection to the fragile PZT patches. The dimension of each PZT patch is 15 mm × 15 mm × 1 mm. Figure 2b shows the photo of the smart aggregate that was used in the experiment. The height and diameter of the smart aggregates is 20 mm and 25 mm, respectively.

**Figure 3:**
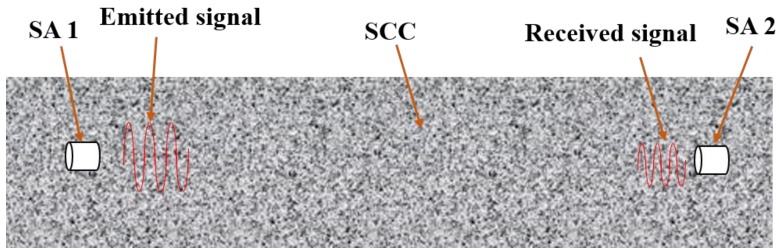
The schematic of smart aggregate based active sensing approach.

Owing to the piezoelectric properties of the PZT, smart aggregate can work both as an actuator and a sensor. In this research, the active sensing approach was utilized. The schematic of the active sensing approach is shown in Figure 3.

There are two SAs in each SCC specimen. SA1 works as an actuator and SA2 works as a sensor. A sweep sine signal (100–100k Hz) is emitted by SA1. The period of the sweep sine signal in this research is 1 s. When the emitted sweep signal propagates to the SA2, the signal is detected by the sensor. As the hydration progresses, the received signal changes in both time and frequency domains. By analyzing the received signals, the hydration characterization of SCC can be achieved.

## 3. Wavelet Packet Energy Analysis

The hydration of SCC significantly affects the stress wave propagation in both time and frequency domains [40]. The hydration characterization in the early age should be quantitatively evaluated. Wavelet packet energy analysis is extensively used in structural health monitoring [42,43]. By integrating the wavelet-based signal processing with an active sensing system, an online monitoring system for composite structures was established [44]. Du et al. [45] developed a wavelet packet-based damage index matrix to evaluate crack damages on a pipeline structure. The wavelet packet analysis was applied by Zhang et al. [46] to monitor the looseness of the cuplock scaffolds connections.

In the process of early-age hydration monitoring, the received signal was recorded every half hour during the first 24 h. The received signal at *k*th (*k* = 0, 1, 2, …, 48) half hour can be expressed as *H_k_*. It should be notes that *k* = 0 represents the beginning of hydration. By an *n*-level wavelet packet energy decomposition, the received signal *H_k_* is decomposed to *n* + 1 signal sets in different frequency ranges {*S*_1_*^k^*}, {*S*_2_*^k^*}, …, {*S_n_^k^*}, {*S_n+_*_1_*^k^*}. Each signal sets can be expressed as *X_ij_^k^*,

*X_ij_^k^* = [*X_i1_^k^*, *X*_*i*2_*^k^*, *X*_*i*3_*^k^*, …, *X_im_^k^*] (1)

where *i* = 1, 2, …, *n* + 1, *j* = 1, 2, …, *m*, and *m* is the number of samples in each set [47]. The energy of each set of {*S*_1_*^k^*}, {*S*_2_*^k^*}, …, {*S_n_^k^*}, {*S_n+_*_1_*^k^*} can be calculated by,

(2) $$E_{i}^{k}=\sum_{j=1}^{m}{\left|X_{ij}^{k}\right|}^{2}$$

the energy of received signal at *k*th half hour *H_k_* is defined as:

(3) $$E_{H}^{k}=\sum_{i=1}^{n+1}E_{i}^{k}$$

where $E_{H}^{k}$ represents the energy of *H_k_*.

Based on the $E_{H}^{k}$, a hydration completion index (HCI) was proposed as following:

(4) $$HCI_{l}=\sum \frac{E_{H}^{k+1}}{E_{H}^{k}}$$

where $HCI_{l}$ (*l* = *k* + 1 = 1, 2, 3, …, 48) is the hydration completion index at different hydration periods. Attentions should be paid to *k* = 0, which means that it is the completion at the beginning of hydration. In order to show the result more clearly, hydration completion index (HCI) is normalized,

(5) $$NHCI_{l}=\frac{HCI_{l}-HCI_{1}}{HCI_{48}-HCI_{1}}$$

where $HCI_{1}$ and $HCI_{48}$ are the calculated hydration completion indices at the first (0.5 h) and the last (the 24th hour) received signal, respectively. $NHCI_{l}$ presents the normalized hydration completion index (NHCI) at *l*th half hour (*l* = 1, 2, 3, …, 48). Based on the proposed novel normalized hydration completion index (NHCI), the hydration characteristic during different hydration periods can be quantitatively and clearly revealed.

## 4. Specimens, Experiment Setup, and Procedures

### 4.1. SCC Concrete Specimens

#### 4.1.1. Materials

##### Cement

The cement used in this research is composite Portland cement provided by Huarun Cement Manufactory Co., Ltd. located in Dongguan, China. It is of grade 32.5 and the specific surface is 381 m^2^/kg. The chemical composition of the cement is given in Table 1.

##### Fly Ash

Type F Fly ash of class II was used in this experimental study. The chemical composition of fly ash determined by X-ray Fluorescence (XFR) is given in Table 1.

##### Coarse Aggregate

**Figure 4:**
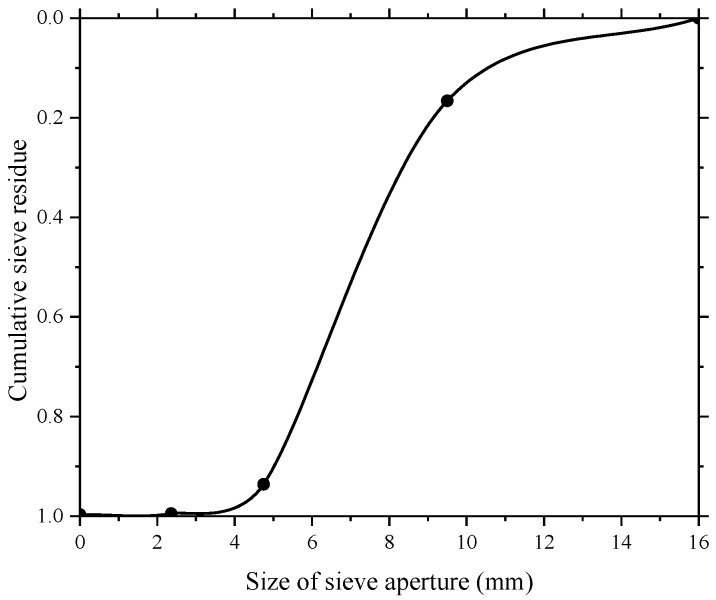
Sieve curve of coarse aggregate.

The particle size of the coarse aggregate used in this test composed of crushed stone was distributed from 5 to 15 mm. The bulk density and apparent density of the coarse aggregate in this experiment were 1352.67 kg/m^3^ and 2579.26 kg/m^3^, respectively. Their particle size distributions are shown in Table 2 and the sieve curve of coarse aggregates is shown in Figure 4.

##### Fine Aggregates

**Figure 5:**
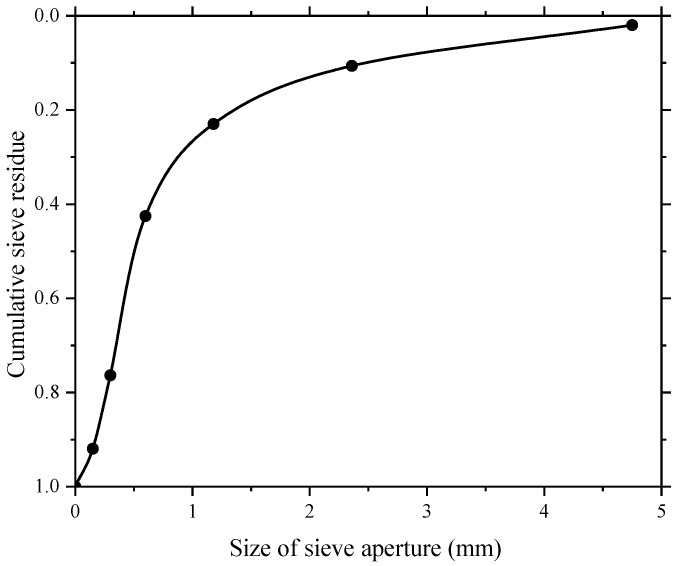
Sieve curve of fine aggregate.

The maximum size of fine aggregate (river sand) used in this study was around 5 mm. The bulk density and apparent density of the fine aggregate were 1571.67 kg/m^3^ and 2666.76 kg/m^3^ respectively. The fine aggregate in the experiment test belongs to medium sand owing to their fineness modulus of 2.39 provided by the sieve test result. The sieve test result and the sieve curve of fine aggregate are given in Table 2 and Figure 5, respectively.

##### Limestone Powder

The cohesiveness of concrete is effectively improved due to the incorporation of limestone powder. The limestone powder with a fineness of 250 meshes were used in this test.

##### Superplasticizer

A type of superplasticizer coded as Melflux2651F, supplied by BASF, was used in this research for ameliorating the working performance of concrete.

#### 4.1.2. Detailed Mix Proportions

The mix details for all four SCC mixtures are summarized in Table 3. The total blinder content was maintained as a constant mass of 42.264 kg and the fly ash was used to replace blinder from 0% to 60% at a 20% interval. The water-to-blinder ratio and limestone powder-to-blinder ratio of all mix proportions were kept constant at 0.343 and 0.137, respectively.

#### 4.1.3. Mixing Process

**Figure 6:**
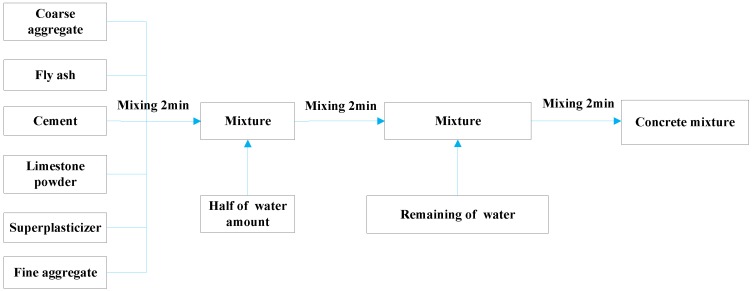
Mixing process of concrete.

The total time of SCC mixing is recommended to be approximately 6 min in this test. Firstly, the coarse aggregate, powder material (fly ash, cement, limestone powder, superplasticizer) and fine aggregate were poured into the concrete-mixer in sequence and stirred for 2 min. Then, the first half of water was added slowly and the concrete was mixed for 2 min. Subsequently, the remaining water was mixed into the concrete-mixer and the mixing process was carried out for about 2 more minutes. The mixing process for all the SCC specimens is shown in Figure 6.

#### 4.1.4. Flesh Properties of Concrete Mixture

**Figure 7:**
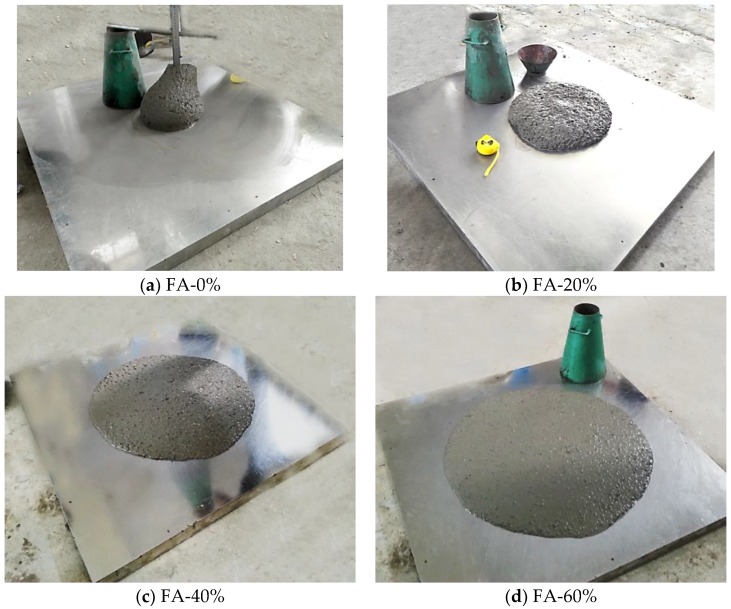
Slump flow tests of the SCC concrete mixture.

A slump flow test for all the SCC mixtures with different volumes of fly ash were conducted (see Figure 7) with the aim to investigate fresh state performance of SCC mixtures. The values of T_500_ (the time required for the concrete mixture flows to the 500 mm mark of the plate), slump and slump flow were obtained by a slump flow test. Additionally, the workability of SCC mixtures, such as cohesiveness, bleeding and segregation, was investigated depending on the observation of the mixing specimens. The fresh properties of all the SCC mixtures are listed in Table 4.

**Figure 8:**
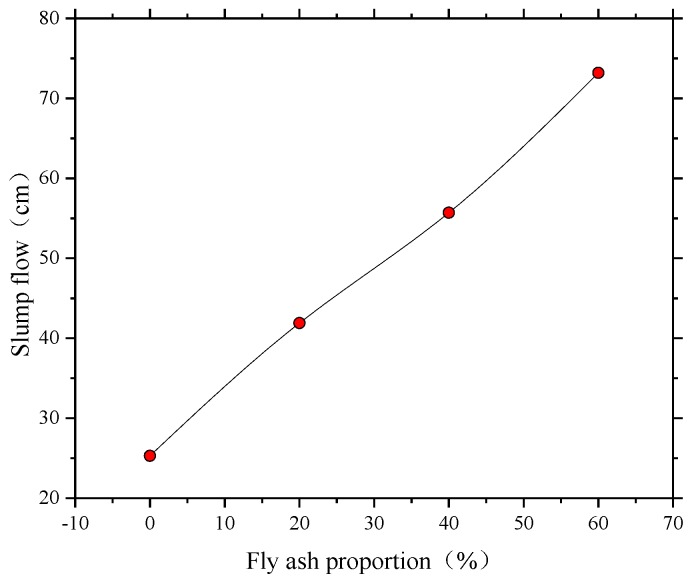
Effect of fly ash proportion on the slump flow.

It is observed that the self-compatibility of concrete mixture reached the requirement of self-compacting concrete when the fly ash proportion exceeded 40% (see Table 4 and Figure 8). The slump flow and T_500_ of SCC are required within the range of 550~850 mm and 2~5 s specified by the European guidelines [48]. All the SCC specimens had sufficient workability for construction. However, the cohesiveness was slightly low for FA-60%, which may be due to using a large volume of fly ash. In this test specimen, the use of high-volume fly ash resulted in a significant ball-bearing effect, which creates a lubricating action in SCC and increases the flow ability. Figure 8 shows that the flow ability of mixtures is greatly improved with the increase in fly ash volume, which implies that fly ash plays a significant role in the fresh properties of SCC.

### 4.2. Test Setup

#### 4.2.1. Active Sensing Using Piezoceramic Transducers

**Figure 9:**
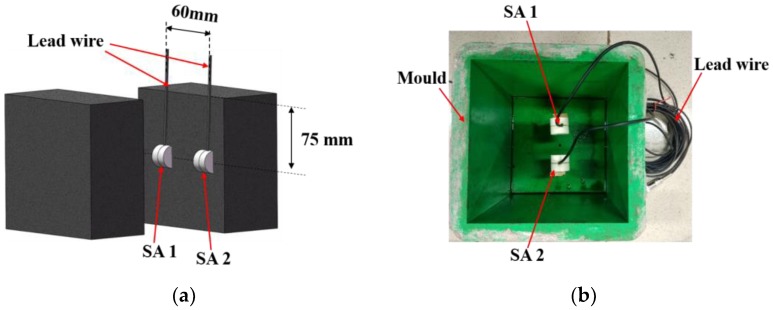
Configuration of SAs in concrete matrix. (**a**) Position of SAs in concrete cube, (**b**) Configuration of SAs in concrete test specimens before casting.

The configuration of SAs in concrete matrix is shown in Figure 9. Two piezoceramic smart aggregates were used in the SCC specimen. They were 75 cm below the top surface of the SCC concrete specimen and the spacing is 60 cm.

**Figure 10:**
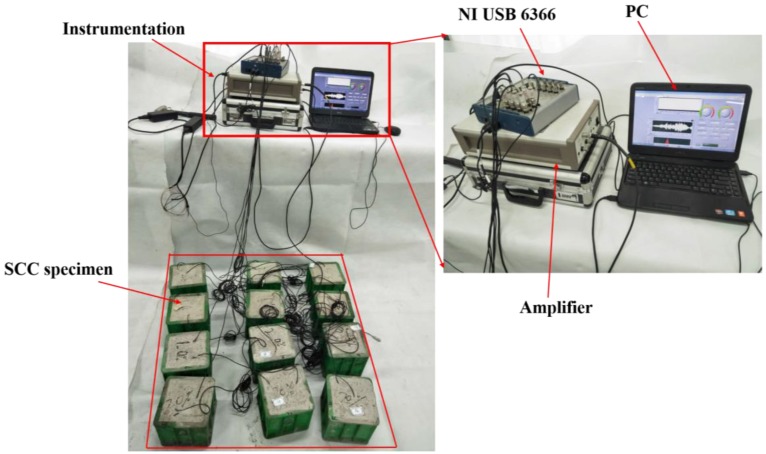
Instrumentation for smart aggregate (SA) based active sensing approach.

The instrumentation used in this experimental test is shown in Figure 10. A data acquisition device (NI-USB 6366) was adopted to generate and receive the signals and a power amplifier was used to amplify the emitted signal.

The sampling rate of the NI-USB 6366 used in this study is 1 MHz. The sweep sine signal from 100 Hz to 100 kHz was selected. The signal period is 1 s. Detailed information about the emitted signal is shown in Table 5.

#### 4.2.2. Penetration Resistance Test

**Figure 11:**
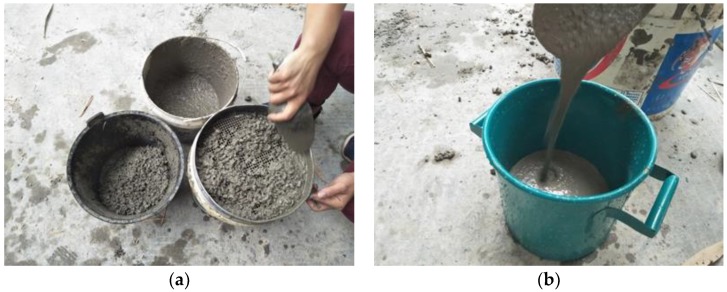
The procedure before the penetration resistance test. (**a**) Coarse aggregate was removed; (**b**) remaining mortar was poured into the barrel.

**Figure 12:**
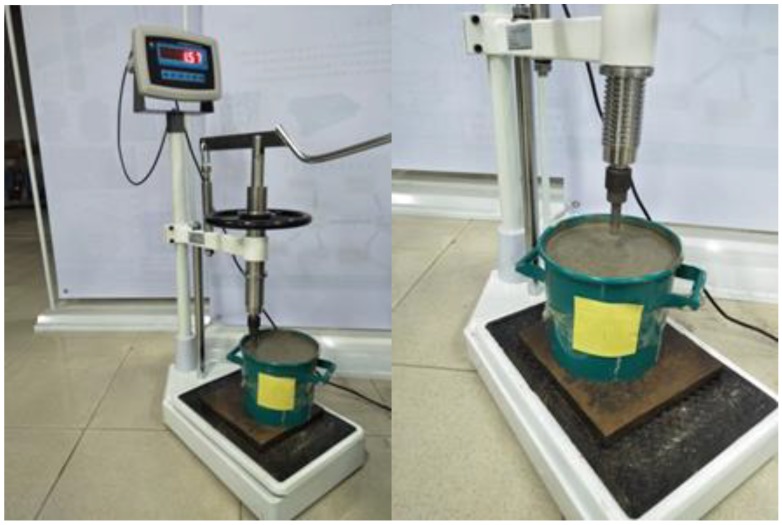
Penetration resistance test.

In this study, a series of experimental tests to achieve penetration resistance of SCCs specimens were carried out. The aim of this experimental study was to determine the initial and final setting time of SCC specimens. The results of penetration-resistance tests were compared with those identified by the signals from SAs. The curve of penetration resistance-time was found by the penetration resistance test, which also implied the early-aged hydration process of the mixture. The initial and final setting times were determined by the corresponding time when the penetration resistance reached 3.5 MPa and 28 MPa, respectively. As shown in Figure 11, the coarse aggregate of the mixture was removed with a sieve of 4.75 mm before the penetration-resistance testing and the remaining mortar was poured into the test barrel. The penetration-resistance testing (see Figure 12) was performed at every hour after the casting. Additionally, as the resistance increased close to 3.5 MPa (initial setting) or 28 MPa (final setting), the interval of the resistance testing was varied to be 0.5 h.

## 5. Experimental Results and Discussion

### 5.1. The Test Results Based on SAs

The hydration performance of SCC with a different volume of fly ash was monitored for 24 h. Utilizing the active sensing approach, the sensor continuously received the propagated wave signal transmitted from the actuator SA. In this study, the collected response signal presented a different amplitude variation trend in the time domain during the stage of SCC transition from liquid state to hardened state. By studying the characteristics of the obtained signals, the hydration performance of SCC was classified.

**Figure 13:**
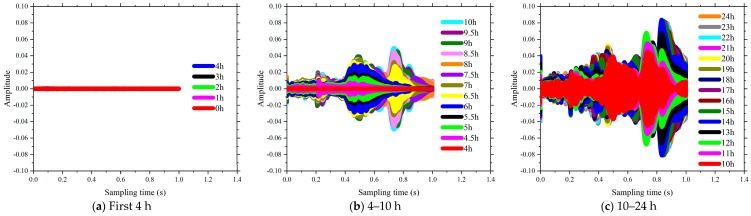
Influence of volume of fly ash on the signal amplitude at hydration monitoring (FA-0%).

**Figure 14:**
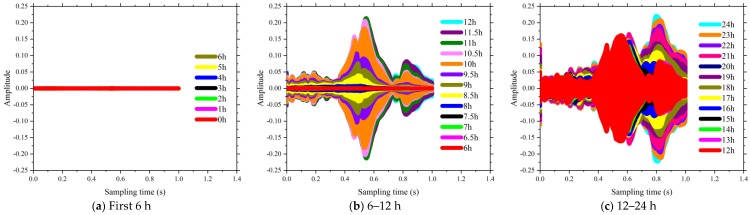
Influence of volume of fly ash on the signal amplitude at hydration monitoring (FA-20%).

**Figure 15:**
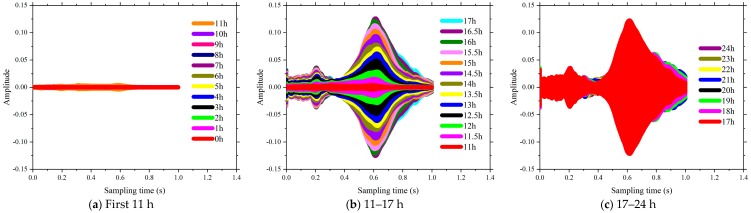
Influence of volume of fly ash on the signal amplitude at hydration monitoring (FA-40%).

**Figure 16:**
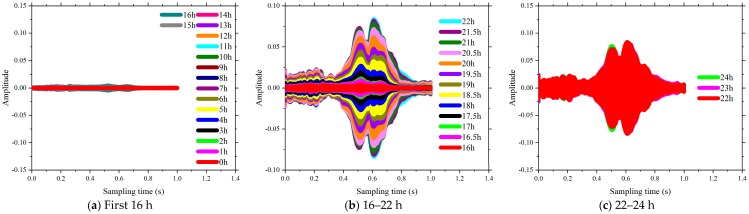
Influence of volume of fly ash on the signal amplitude at hydration monitoring (FA-60%).

The time domain of signal response of SCC in the first 24 h of the curing is shown in Figure 13, Figure 14, Figure 15 and Figure 16. The time interval in this phase of the test was configured as 0.5 h. Each plot presents a complete period sensor signal according to the excitation swept sine wave. It can be found that the amplitudes of the collected sensor signal wave were very weak at the beginning stage for each test specimen. As shown in Figure 13a, all the voltage signals of test specimen coded as FA-0% measured by the SAs were almost the same before the curing time reached the 4th hour. As the curing time increased, the amplitudes of the collected signals were enhanced and the shapes of those signals changed subsequently. For the same specimens of FA-20%, the amplitudes of those signals changed dramatically at around 4.5 h after the casting (see Figure 13b). As shown in Figure 13, Figure 14, Figure 15 and Figure 16, all these signal responses were enhanced suddenly when the curing time reached a certain moment. This indicates that the energy propagating through the concrete between the two SAs was improved significantly at this moment as curing time went on. This moment can be regarded as the initial setting time of SCCs when the status of concrete started to be transited from liquid stage to hardened stage. Interestingly, the time corresponding to this significant enhancement in the signal response was delayed by increasing the volume of fly ash as replacement of cement. It can therefore be concluded that increasing the volume of fly ash in SCC influenced the speed of the formation of the microstructure and resulted in a longer initial setting time, such as 4 h for FA-0%, 8 h for FA-20%, 11 h for FA-40% and 16 h for FA-60%. This could be attributed to using a high-volume of fly, as the replacement of cement reduced the rate of heat production, lengthened the dormant period and delayed the start of pozzolanic activity [49]. Thereafter, as the hardening developed inside the matrix of SCC, the stress wave transmission through SCCs became more intense and the maximum value of voltage signals were enhanced significantly (see Figure 13, Figure 14, Figure 15 and Figure 16). At the same time, the strength of SCCs increased rapidly though this stage.

It can be seen in Figure 13, Figure 14, Figure 15 and Figure 16 that the voltage values in the signal responses keep increasing after this initial setting moment, which is due to the strong development of the hydration reaction in SCCs. This stage is classified as an SCC transition stage from the liquid stage to the hardened stage. In addition, this significant variation of the signal response was terminated at a certain moment, such as 7 h for FA-0%, 11 h for FA-20%, 16 h for FA-40% and 21.5 h for FA-60%. This moment can be considered as the final setting time of SCCs and the end of the transition stage. After this stage, the signals of voltage amplitudes began to present a similar trend, which indicated that the hydration was approaching stability. Meanwhile, with the development of hardening properties of SCCs, more stable and smooth amplitude of the voltage signals were achieved (see Figure 13, Figure 14, Figure 15 and Figure 16). It can be summarized that the duration of the transition stage was around 3 to 5 h for all the test specimens and increasing the volume of fly ash resulted in longer durations of transition stages. This also indicates that the increase of fly as in SCC mixing resulted in less intensity of the hydration [50]. As a result, the early strength of SCC was subsequently reduced by increasing the usage of fly ash in concrete mixing, as shown in Figure 1.

### 5.2. The Results of Penetration Resistance Test

**Figure 17:**
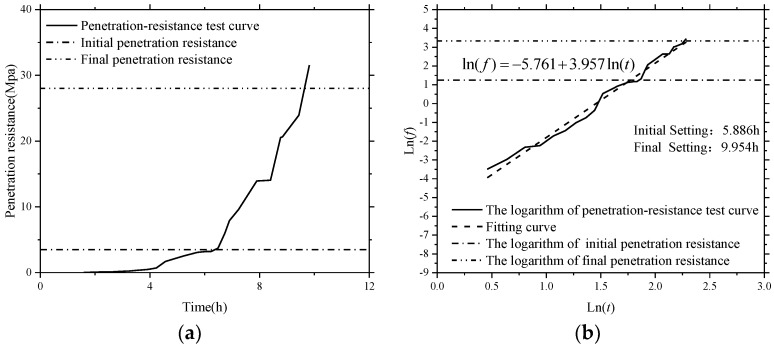
Typical penetration-resistance test results of SCC with 0% fly ash. (**a**) Penetration-resistance test curve; (**b**) the logarithm of penetration-resistance test curve.

**Figure 18:**
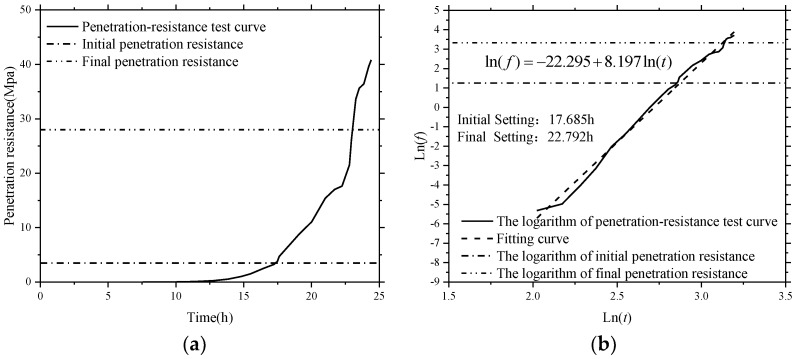
Typical penetration-resistance test results of SCC with 60% fly ash. (**a**) Penetration-resistance test curve; (**b**) the logarithm of penetration-resistance test curve.

To investigate the feasibility of using the active sensing approach based on SAs to monitor the hydration performance of SCCs with different volume of fly ash, the initial and final setting times of all the test specimens were determined by a series of penetration resistance tests presented above. According to the Chinese Standard [51], the initial and final setting times of SCC were determined primarily when penetration resistance of a sieved mortar sample (see Figure 12) reached 3.5 N/mm^2^ and 28 N/mm^2^, respectively (see Figure 17 and Figure 18). Therefore, the accurate setting times of SCCs were predicted through a data fitting process, as shown in Figure 17 and Figure 18. The initial and final setting times determined by penetration resistance test for all the SCC test specimens in this study are presented in Table 6. It can be found that the initial and final setting times of SCC were delayed by increasing the percentage of fly ash as replacement for cement.

### 5.3. Correlations of the Results from the Monitoring Based on SAs and the Penetration Resistance Tests

As shown in Table 6, the initial and final setting times predicted by signals from SAs were compared to those obtained by the penetration resistance test. It can be seen that a good correlation of the results between the active sensing approach and the mechanical test is obtained in this comparative study. This suggests that the active sensing approach based on SAs can be used to investigate the hydration characteristics of SCCs with different volumes of fly ash accurately. Interestingly, the results from the monitoring based on SAs and the penetration resistance tests both revealed that the duration between the initial and final times was extended by increasing the volume of fly ash. This suggests that a larger volume of fly ash as a replacement for cement resulted in a longer transition stage for SCCs.

**Figure 19:**
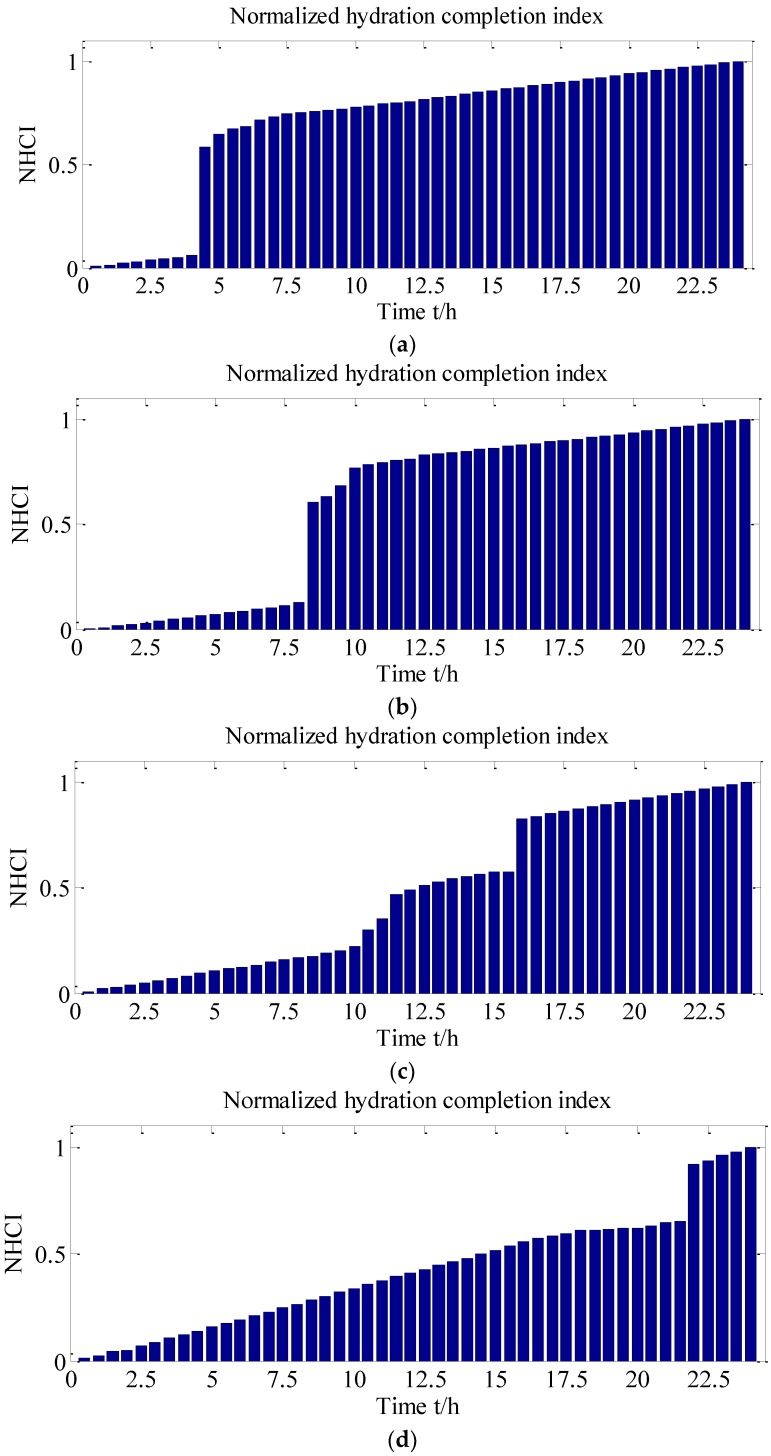
NHCI for SCCs with different volume of fly ash. (**a**) NHCI for FA-0%; (**b**) NHCI for FA-20%; (**c**) NHCI for FA-40%; (**d**) NHCI for FA-60%.

**Figure 20:**
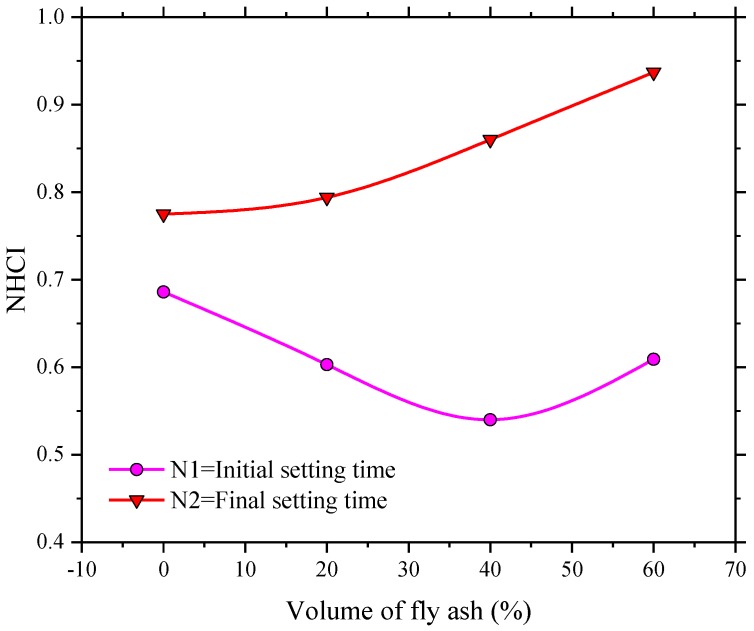
Influence of volume of fly ash in SCCs on NHCI values at initial and final setting times.

**Figure 21:**
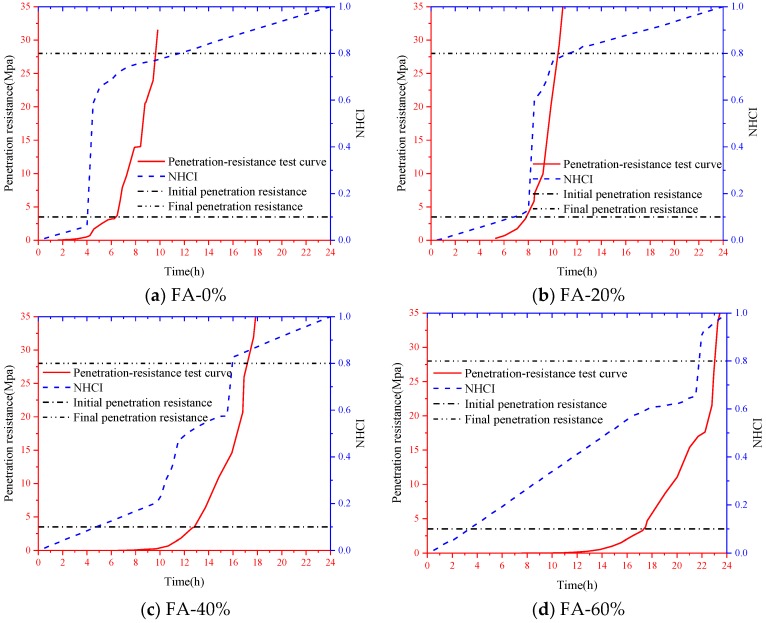
Comparison of the development process of NHCI values and the test results penetration resistance in the first 24 h after the casting of SCCs.

However, the single amplitudes of those voltages provided by SAs were not sufficiently precise and convenient to identify the hydration performance of SCCs. From Figure 13, Figure 14, Figure 15 and Figure 16, the voltage amplitude of the received signal increased gradually within 24 h. To develop a quantitative and accurate study of hydration performance of SCCs with different-volume fly ash, a wavelet packet energy analysis method was used to predict the normalized hydration completion index (NHCI). Based on the proposed normalized hydration completion index (NHCI), the monitoring results of the SCC with fly ash of 0%, 20%, 40%, 60% are listed as following:

The proposed NHCI, as shown in Figure 19, clearly revealed the hydration characteristics during different hydration stages. It can be found that the NHCI values of all the SCC specimens were increased dramatically at the initial setting time corresponding to the test results shown in Table 6. Taking an example of FA-0%, for the beginning of 4 h, the NHCI of the self-compacting concrete without fly ash is much small and it is lower than 0.1. However, the hydration speed was increased rapidly between the 4th to the 5th hour, particularly at 4.5 h, which should be corresponding to the initial setting time of this test specimen. The NHCI in Figure 19 clearly indicates the hardening process of the SCC. Similar conclusions can be draw from Figure 12 and Table 5. As shown in Figure 19, NHCI values indicate that with 0%, 20%, 40%, 60% fly ash, the hydration mainly increases at 4.5, 8.5, 11.5, and 18th hour. The rapid increase of NHCI at those moments also means that the starting age of transition stage of SCCs with different-volume fly ash. This enhancement degree of NHCI values was decreased after the final setting time was reached. Subsequently, the variation of NHCI value was steady and stable, which indicated the status of SCCs were transferred to be hardened. Figure 19 also illustrated that increasing the volume of fly ash delayed a start of the setting as well as reduced hydration speed [52]. In addition, the NHCI values at initial and final setting times predicted by the signals provided by SAs are illustrated in Figure 20. It was shown that the difference between N2 (NHCI at final setting) and N1 (NHCI at initial setting time) increased with the percentage of fly ash as replacement of cement in SCCs. This graphically indicates that increasing the volume of fly ash resulted in longer durations of transition stages from liquid status to hardened status of SCCs, which is corresponding to the investigation results shown in Table 5. The development process of NHCI values and the test results of penetration resistance of SCCs were compared, as shown in Figure 21. It can be noted that the development of hydration performance in the first 24 h predicted by NHCI had reasonable agreement with the test results of penetration resistance of SCCs. Therefore, it can be concluded that the effect of fly ash on early-age hydration characteristics in SCC was effectively revealed by the proposed NHCI approach.

## 6. Conclusions

A feasibility study of using a piezoceramic-transducer-enabled active sensing approach to investigate the hydration performance of self-compacting concrete (SCC) with different volumes of fly ash is carried out in this research. The very early-age (the first 24 h after casting) hydration characteristics of a series of SCC specimens were monitored by piezoceramic transducers, which enabled the active sensing approach. By investigating the amplitude of the propagated wave between a pair of embedded smart aggregates (SAs), the hydration characteristics, particularly in the transition stage of SCCs from liquid state to hardened state, were verified clearly. This paper opens a door to the investigation of the influence of high-volume fly ash on the hydration effect of self-compacting concrete using piezoceramic transducers. The accuracy of this novel test method was validated by the comparison of the test results by penetration test. Additionally, a normalized hydration completion index (NHCI) was proposed by a wavelet packet analysis to determine the initial and final setting time accurately and quantitatively. The main findings and conclusions in this paper are summarized as below:

(1) It is evident in the test results that using a high volume of fly ash as a replacement for cement has clear effects on the formation of the microstructures in SCC. Replacing cement by fly ash in SCC mixing decelerates the completion of hydration, which is attributed to the delayed start of the pozzolanic activity in microstructure of SCC.
(2) By studying the amplitude of the propagated wave between a pair of embedded SAs, three hydration stages were clearly verified. In the liquid stage, the microstructure of the SCC changed steadily. The response of the signal provided by SAs was correspondingly stable. As the SCC entered the transition stage, the properties were changing dramatically. Thus, the voltage signals were enhanced significantly and the stress wave transmission through the hardening SCC became more intense. As a result, those signal responses were increased significantly after the initial setting. From the final setting time (hardened stage) and onwards, the hydration turned to the hardened stage and the amplitudes of the voltage signals became stable and steady, due to the smooth reaction in the microstructure of SCC.
(3) The results of initial and final setting time of SCC test specimens predicted by the signal based on SAs showed good agreement with the results obtained from a series of penetration-resistance experimental tests. The results from those two experimental tests also revealed that the duration of the transition stage of SCCs were extended by increasing the volume of fly ash.
(4) To quantitatively evaluate the hydration completion of SCCs with different volumes of fly ash, a normalized hydration completion index (NHCI) was proposed through a wavelet packet analysis. Based on the validation of the NHCI values with the test results of the penetration resistances, it was found that this index could offer an accurate assessment of the early-age hydration performance of SCCs.

In summary, the proposed test method is convenient and offers a real-time tool to investigate the concrete materials with high-volume industrial by-product. The investigation results can be used to explain the hydration and strength development of self-compacting concrete with high-volume fly ash. The research achievement is beneficial to developing sustainable construction materials by using industrial by-products to replace cement.

## Figures and Tables

**Table 1:** Chemical Composition of Fly ash and Cement.

Compound	SiO_2_ (%)	Fe_2_O_3_ (%)	CaO (%)	K_2_O (%)	SO_3_ (%)	TiO_2_ (%)	MnO_2_ (%)	SrO (%)
Fly ash	60.7	18.7	10.2	4.3	-	4.1	0.4	0.7
Cement	13.8	6.0	75.7	-	3.7	0.4	0.2	-

**Table 2:** Particle size distribution of coarse aggregates.

Coarse Aggregate		Fine Aggregate	
Size of Sieve Aperture (mm)	Cumulative Sieve Residue (%)	Size of Sieve Aperture (mm)	Cumulative Sieve Residue (%)
19.00	0.0	4.75	2.928
16.00	0.0	2.36	11.416
9.50	16.6	1.18	23.662
4.75	94.4	0.60	42.976
2.36	99.8	0.30	76.904
bottom plate	100.0	0.15	92.036
		bottom plate	99.820

**Table 3:** Mix details of SCCs in this test.

Test Specimen	Coarse Aggregate (kg)	Fine Aggregate (kg)	Fly Ash (kg)	Cement (kg)	Limestone Powder (kg)	Water (kg)	Superplasticizer (%)
FA-0%	56.183	56.611	0.000	36.479	5.785	14.513	0.16
FA-20%	56.183	56.611	8.453	28.026	5.785	14.513	0.16
FA-40%	56.183	56.611	16.906	19.573	5.785	14.513	0.16
FA-60%	56.183	56.611	25.358	11.121	5.785	14.513	0.16

**Table 4:** Flesh properties of concrete mixture.

NO.	T_500_/(s)	Slump/(cm)	Slump Flow/(cm)	Cohesiveness	Bleeding	Segregation
FA-0%	-	11.7	25.3	Good	Not occurred	Not occurred
FA-20%	-	25.1	41.9	Good	Not occurred	Not occurred
FA-40%	3	-	55.7	Good	Not occurred	Not occurred
FA-60%	2	-	73.2	General	Not occurred	Not occurred

**Table 5:** Detailed properties of emitted signal.

Start Frequency (Hz)	Stop Frequency (kHz)	Amplitude (V)	Duration (s)
100	100	3	1

**Table 6:** Comparison of initial and final setting time obtained by SAs and penetration test.

Specimen	Initial Setting		Final Setting	
	SAs	Penetration Test	SAs	Penetration Test
FA-0%	4	5.9	7	10.0
FA-20%	8	7.8	11	10.8
FA-40%	11	13.2	16	17.4
FA-60%	16	17.7	21.5	22.8
